# Susceptibility of *Staphylococcus aureus* to Anti-Inflammatory Drugs with a Focus on the Combinatory Effect of Celecoxib with Oxacillin In Vitro

**DOI:** 10.3390/molecules29153665

**Published:** 2024-08-02

**Authors:** Onyedika Emmanuel Okpala, Johana Rondevaldova, Hayford Osei-Owusu, Tomas Kudera, Tersia Kokoskova, Ladislav Kokoska

**Affiliations:** 1 Department of Crop Sciences and Agroforestry, Faculty of Tropical AgriSciences, Czech University of Life Sciences Prague, Kamycka 129, Suchdol, 165 00 Prague, Czech Republic; okpala@ftz.czu.cz (O.E.O.); rondevaldova@ftz.czu.cz (J.R.); osei-owusu@ftz.czu.cz (H.O.-O.); 2 Drift-Food Research Centre, Faculty of Agrobiology, Food and Natural Resources, Czech University of Life Sciences Prague, Kamycka 129, Suchdol, 165 00 Prague, Czech Republic; kuderat@af.czu.cz; 3 Department of Animal Science and Food Processing, Faculty of Tropical AgriSciences, Czech University of Life Sciences Prague, Kamycka 129, Suchdol, 165 00 Prague, Czech Republic; kokoskova@ftz.czu.cz

**Keywords:** antibacterial activity, antistaphylococcal synergistic effect, methicillin-resistant *S. aureus*, musculoskeletal infections, non-steroidal anti-inflammatory drugs

## Abstract

Musculoskeletal infections (MIs) are among the most difficult-to-treat staphylococcal diseases due to antibiotic resistance. This has encouraged the development of innovative strategies, such as combination therapy, to combat MI. The aim of this study was to investigate the in vitro antistaphylococcal activity of anti-inflammatory drugs and the combined antimicrobial effect of celecoxib and oxacillin. The minimum inhibitory concentrations (MICs) of 17 anti-inflammatory drugs against standard strains and clinical isolates of *S. aureus*, including methicillin-resistant strains (MRSAs), were determined using the broth microdilution method. The fractional inhibitory concentration indices (FICIs) were evaluated using checkerboard assays. Celecoxib produced the most potent antistaphylococcal effect against all tested strains (MICs ranging from 32 to 64 mg/L), followed by that of diacerein against MRSA3 and MRSA ATCC 33592 (MIC 64 mg/L). Several synergistic effects were observed against the tested *S. aureus* strains, including MRSA (FICI ranging from 0.087 to 0.471). The strongest synergistic interaction (FICI 0.087) was against MRSA ATCC 33592 at a celecoxib concentration of 2 mg/L, with a 19-fold oxacillin MIC reduction (from 512 to 26.888 mg/L). This is the first report on the combined antistaphylococcal effect of celecoxib and oxacillin. These findings suggest celecoxib and its combination with oxacillin as perspective agents for research focused on the development of novel therapies for MI caused by *S. aureus.* This study further indicates that celecoxib could resensitize certain MRSA strains, in some cases, to be susceptible to β-lactams (e.g., oxacillin) that were not previously tested. It is essential to mention that the in vitro concentrations of anti-inflammatory drugs are higher than those typically obtained in patients. Therefore, an alternative option for its administration could be the use of a drug delivery system for the controlled slow release from an implant at the infection site.

## 1. Introduction

*Staphylococcus aureus* is part of the normal human flora; about 30% of the population harbors nasal *S. aureus*, and those patients are typically asymptomatic [1,2]. On the other hand, *S. aureus* has become a dangerous human pathogen, causing various infections ranging from skin and soft tissue infections (SSTIs) to severe septicemia [3]. Staphylococcal infections are commonly treated with antibiotics; however, many strains are resistant to previously effective treatments. A previous study showed that more than 150,000 patients are infected with methicillin-resistant *S. aureus* (MRSA) yearly in the EU, causing an additional cost of EUR 380 million to the EU healthcare system [4].

Musculoskeletal infections (MIs) are one of the most difficult-to-treat staphylococcal diseases affecting the bone and surrounding tissues [5,6]. Although several other microorganisms, including fungi (e.g., *Exophiala dermatitidis*) and certain viruses (e.g., *Varicella zoster*), have been identified as causal agents of MI [7,8], *S. aureus* is the principal bacterial agent responsible for the most severe forms of MI, such as osteomyelitis and septic arthritis [9]. Both of these conditions are associated with patient morbidity and mortality [10,11]. Epidemiological data from Germany showed an osteomyelitis incidence rate of 17 cases per 100,000 inhabitants [12]. At the same time, four to ten infections per 100,000 patients per year were reported for septic arthritis, with a high mortality rate of 10–15% [13,14].

Antibiotic therapy and surgical debridement are the main treatment options for osteomyelitis and septic arthritis [15,16]. Surgical debridement, a procedure that involves removing necrotic bone and irrigating abscessed tissue, is used to reduce the bacterial burden and enhance antibiotic delivery [17,18,19]. β-Lactams (e.g., oxacillin) are the preferred choice for treating methicillin-susceptible *S. aureus* (MSSA) osteomyelitis compared to antibiotics, such as glycopeptides (vancomycin), commonly used in treating MIs, such as MRSA osteomyelitis [20]. For example, clinical trials involving patients with MIs caused by MSSA infection demonstrated that oxacillin treatment resulted in 92% clinical success [21]. Furthermore, this drug is used primarily as an antistaphylococcal agent for treating suspected hematogenous osteomyelitis in children with mild to moderate infections, especially in regions with low rates of community-acquired MRSA osteomyelitis [22]. For this reason, oxacillin is among the preferred drugs for treating MIs (e.g., infectious osteomyelitis) caused by MSSA [20]. Although vancomycin is the first-line agent for treating invasive MRSA infections, it is associated with poorer tissue penetration, nephrotoxicity, ototoxicity, and thrombocytopenia [23,24,25]. Clindamycin, while effective against MSSA, may lead to cross-resistance and inducible resistance in MRSA. It is also associated with a high resistance rate of >20% and *Clostridioides difficile* infection. Conversely, oxacillin is recommended by the Infectious Diseases Society of America as a parenteral drug choice for the treatment of MSSA-induced SSTIs [18,26,27]. Despite the benefits of using oxacillin for the treatment of MIs, MRSA, which causes chronic MI (e.g., septic arthritis), remains a severe clinical problem [28,29]. The combination of antimicrobial agents may be an option for the treatment of MIs caused by MRSA strains. In general, combination therapy may be beneficial due to the reduced concentration required for each drug used (with reduced side effects) and the delayed development of drug resistance because of the multiple targeting mechanisms of the combined drugs [30]. For example, the combination of isoniazid, rifampicin, ethambutol, and pyrazinamide, which target diverse pathways, is used for the treatment of tuberculosis [31,32], while the drug cotrimoxazole comprises sulfamethoxazole and trimethoprim, which are two medicines that function at various stages within a single pathway to achieve better inhibition than using either of those drugs alone. This specific targeting reduces drug doses required and toxicities, leading to a favorable patient outcome [33]. Due to the serious challenge encountered in treating MRSA-induced MIs with antibiotics, combination therapy involving oxacillin and other agents with different mechanisms of action (e.g., anti-inflammatory drugs) seems to be a promising treatment option to combat these difficult-to-treat infections.

Anti-inflammatory drugs are among the most prescribed medications worldwide [34]. They are generally classified into corticosteroids and non-steroidal anti-inflammatory drugs (NSAIDs). As short-term therapy, corticosteroids are beneficial in treating inflammatory conditions. However, they are not the preferred option for treating chronic conditions due to their association with adverse reactions such as hypertension, osteoporosis, and aseptic joint necrosis, among others [35,36]. Certain corticosteroids, such as corticosterone, have demonstrated antimicrobial properties. Nonetheless, only a few of them show an effect that is inferior to that of currently used antimicrobial agents [37]. In contrast, the antimicrobial potency of NSAIDs (e.g., celecoxib and diclofenac) against certain bacteria, such as *S. aureus* and *S. epidermidis*, has been previously demonstrated in several studies [38,39,40]. Although NSAIDs (e.g., celecoxib) are generally considered to be efficient and well-tolerated drugs for treating chronic inflammatory conditions, non-selective COX inhibitors, such as naproxen, may cause gastrointestinal damage in some patients [41,42]. On the contrary, selective COX-2 inhibitors typically reduce the risk of serious gastrointestinal toxicity. However, it must be mentioned that certain selective COX-2 agents, such as rofecoxib, were associated with an increased risk of serious cardiovascular events. In addition, celecoxib therapy may also result in an elevated risk of heart-related problems; however, this may only occur when used at a dose substantially higher than recommended for treating arthritis [43,44]. Celecoxib is a selective COX-2 inhibitor approved for treating rheumatoid arthritis, which is one of the most common risk factors associated with MI [45,46,47]. It is well established in clinical practice that besides using antibiotics to control bacterial infections, patients often consume anti-inflammatory drugs (e.g., NSAIDs) to treat the inflammation associated with these infections [48]. For example, a clinical study shows that antibiotics administered combined with anti-inflammatory drugs to patients with chronic osteomyelitis resulted in complete symptom resolution [49]. Since celecoxib has already been used in clinical practice [45], it may offer additional benefits to MI patients. The synergistic effects of celecoxib when used as a topical antimicrobial agent together with conventional antibiotics, such as vancomycin and clindamycin, have been previously demonstrated against *S. aureus* [39]. Other NSAIDs (e.g., aspirin, ibuprofen, and diclofenac) have also exhibited antibacterial activity against certain pathogenic bacteria such as *Bacillus cereus*, *Pseudomonas aeruginosa*, and MRSA. In addition, ibuprofen and aspirin demonstrated a synergistic interaction with cefuroxime and chloramphenicol against MSSA and MRSA reference strains [50]. Despite the above-mentioned studies, research on the antistaphylococcal effects of NSAIDs and their combined effects with other antibiotics have not yet been sufficiently explored. Thus, this study investigates the in vitro growth-inhibitory effect of 17 anti-inflammatory drugs (16 NSAIDs and one corticosteroid) against standard strains and clinical isolates of *S. aureus* and the combined antistaphylococcal effect of celecoxib and oxacillin.

## 2. Results

Among the seventeen anti-inflammatory agents tested in this study, six compounds (celecoxib, diacerein, diflunisal, mefenamic acid, sulindac sulfide, and tolfenamic acid) demonstrated in vitro growth-inhibitory effects against various *S. aureus* strains. Celecoxib showed the most potent antistaphylococcal effect against all the tested strains, including clinical MRSA isolates, with MICs ranging from 32 to 64 mg/L (Table 1), followed by diacerein, which inhibited MRSA3 and ATCC 33592 at a MIC of 64 mg/L and produced moderate activity against all the other strains (MIC = 128 mg/L). In addition, sulindac sulfide and tolfenamic acid displayed moderate to weak effects, with MICs ranging from 128 to 512 mg/L against all strains tested. Similarly, mefenamic acid and diflunisal showed weak to no antistaphylococcal activity (MIC ≥ 512 mg/L). Other compounds tested, namely acetylsalicylic acid, acemetacin, ampyrone, cortisone, diclofenac sodium, ethenzamide, felbinac, ibuprofen, nabumetone, propyphenazone, and sulindac produced no effect on the growth of *S. aureus* (MIC > 512 mg/L). The clinical isolate (MRSA1) was the most susceptible strain to celecoxib treatment, with a MIC of 32 mg/L. The growth-inhibitory effects of the tested anti-inflammatory drugs against *S. aureus* are summarized in Table 1.

The celecoxib/oxacillin combination was chosen as the most promising anti-inflammatory drug/antibiotic combination because celecoxib produced the most potent antibacterial action against all tested *S. aureus* strains compared with all other anti-inflammatory drugs tested in this study, and oxacillin has been reported to perform better than most antibiotics when treating infections caused by MSSA [20]. In this combination, several synergistic effects were observed for almost all the standard *S. aureus* strains (except ATCC BAA 976) and two of the clinical isolates (MRSA1 and MRSA4) tested, with the fractional inhibitory concentration indices (FICIs) ranging from 0.087 to 0.471 (Table 2). The strongest synergistic interactions (FICI 0.087) were obtained against MRSA ATCC 33592 at a celecoxib concentration of 2 mg/L with a 19-fold oxacillin MIC reduction (from 512 to 26.888 mg/L). In addition, there was a consistently strong synergistic effect (FICI 0.087, 0.095, and 0.097) against this strain at a celecoxib concentration ranging from 4 to 0.5 mg/L with 12-fold and 40-fold oxacillin MIC reduction from 512 to 40.888 mg/L (highest reduction) and from 512 to 12.777 mg/L, respectively. Moreover, the MIC of oxacillin against MRSA ATCC 33591, ATCC 33592, and ATCC 43300 decreased from resistant (MICs ranging from 227.56 to 512 mg/L) to susceptible (MICs ranging from 2 to 3.555 mg/L) at a celecoxib concentration of 16 mg/L. Regarding the susceptible strains, celecoxib also showed a relatively strong synergistic effect (FICI 0.369) at a drug concentration of 0.5 mg/L, with a 3-fold antibiotic MIC reduction ranging from 0.25 to 0.090 mg/L against ATCC 25923 and from 0.5 to 0.180 mg/L against ATCC 29213. On the other hand, there was no interaction (FICI ranging from 0.507 to 1.006) between oxacillin and celecoxib against MRSA2, MRSA3, and ATCC BAA 976. No antagonistic effect was observed when celecoxib and oxacillin were combined against all the tested strains. The complete results on the combined effects of oxacillin with celecoxib against *S. aureus* strains are summarized in Table 2.

**Figure 1:**
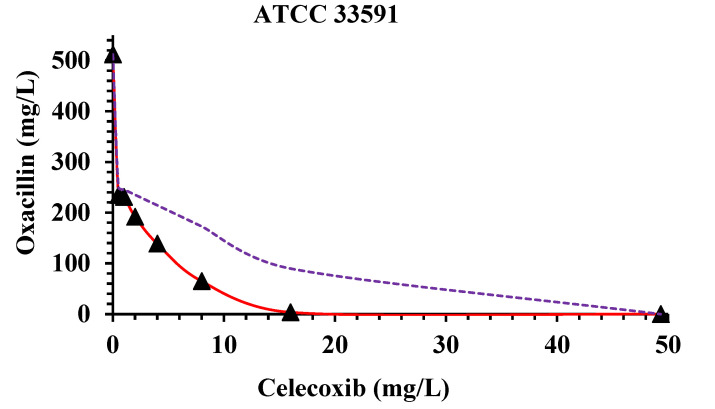

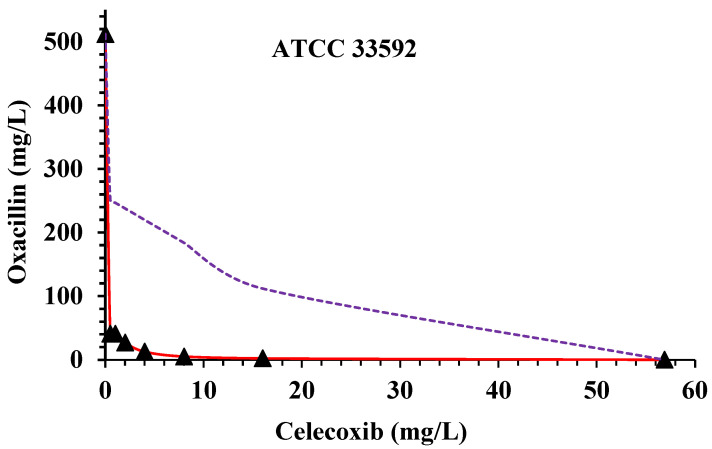
Isobolograms of the most potent synergistic interactions for oxacillin and celecoxib against ATCC 33591 (**top**) and ATCC 33592 (**bottom**). An upward concave curve (dark red solid lines) confirms the antibacterial synergy observed against the *S. aureus* strains, while dotted purple lines represent the synergy border (FICI = 0.5).

The combination profiles of the most susceptible *S. aureus* strains are shown graphically in the form of isobologram curves in Figure 1, representing the FICI values, where the axes of each isobologram are the dose axes of the individual drugs. The figures confirmed the synergistic effect of oxacillin and celecoxib against *S. aureus* strains, where the most robust synergy was observed for six ratios in the isobolograms of MRSA ATCC 33591 and ATCC 33592.

## 3. Discussion

Since available clinical data from randomized controlled trials show no significant differences in efficacy between bactericidal and bacteriostatic agents in the treatment of musculoskeletal infections [3,51,52,53], MIC values, which measure the bacteriostatic action of drugs, were used to evaluate the antibacterial efficacy of the tested anti-inflammatory drugs in the present study. In several previous studies, celecoxib produced in vitro growth-inhibitory effects against standard strains of *S. aureus*. For example, the suppressive effect of this anti-inflammatory drug against *S. aureus* ATCC 29213 and ATCC 33592 at a MIC of 32 mg/L has been previously reported [38]. Furthermore, another study showed the antimicrobial action of celecoxib against MRSA ATCC 4330 at a MIC of 32 mg/L. In addition, the susceptibility assessment of clinical isolates to celecoxib showed MICs ranging from 16 to 128 mg/L [39]. Since the endpoint values determined in the present study lie within the two-dilution range of previously published MICs, these results correspond well with previously published data. Diacerein is a semisynthetic anthraquinone class of anti-inflammatory drugs used for treating osteoarthritis [54,55]. According to a previous study, this drug displayed antistaphylococcal activity against *S. aureus* (susceptible and resistant strains), with the MIC ranging from 16 to 64 mg/L [56]. Additionally, it has been previously demonstrated that diacerein exerted in vitro antimicrobial action against *S. aureus* clinical isolates with the MIC ranging from 4 to 32 mg/L [57]. In the present study, this drug also produced an antibacterial effect against all the *S. aureus* strains tested, including the clinical isolates and MRSA, with an endpoint value within the two-dilution range of previously obtained MICs. Tolfenamic acid is an NSAID and an anthranilic acid drug, usually administered together with antibiotics to treat inflammation and other symptoms associated with bacterial infections [58]. The antibacterial effects of tolfenamic acid have been previously demonstrated against *S. aureus* NCTC8325 at a MIC of 163 mg/L [59], which is similar to the results obtained in the present study. It is well described in the literature that sulindac, a sulfinylindene derivative prodrug, must be converted in vivo to its pharmacologically active metabolite, sulindac sulfide, to act as a non-selective COX inhibitor [60]. Although this agent produced no antimicrobial action in the present study, its metabolite showed a moderate growth-inhibitory effect against all tested *S. aureus* strains. This finding agrees with a previous study that demonstrated the antibacterial effect of sulindac sulfide against *S. aureus* strains (including ATCC 29213) at a MIC of 175 mg/L [61]. In accordance with the breakpoint values indicated in the standards for antibacterial susceptibility testing, the MIC range of oxacillin obtained in this study against the *S. aureus* ATCC 29213 standard strain indicates their susceptibility to oxacillin [62]. The synergistic effect of celecoxib together with various classes of antibiotics has been reported in several studies [39,63]. Celecoxib combined with ampicillin significantly reduced the number of colony-forming units (CFUs) of clinical isolates and the mouse macrophage-phagocytosed standard strain of *S. aureus* when using the agar dilution method and intracellular bacterial viability assay, respectively [63,64]. Nevertheless, this is the first report on the combined antistaphylococcal effect of celecoxib and oxacillin, demonstrating that celecoxib could, in some instances, resensitize certain MRSA strains to become susceptible to β-lactams. 

*S. aureus* reportedly develops resistance to β-lactams through two major mechanisms: using β-lactamases to inactivate the antibiotics and through the production of a low-affinity penicillin-binding protein 2a (PBP2a). For example, a previous report shows that MRSA ATCC 43300, one of the strains used in the present study, expresses PBP2a. The MIC obtained for this strain (20 mg/L) for oxacillin was higher than that of MSSA (2.50 mg/L), confirming the resistance of this strain to the antibiotic [65,66]. The production of PBP2a, an enzyme that provides transpeptidase activity to allow cell wall biosynthesis [67], is the most common mechanism for MRSA development [67]. Under normal circumstances, *S. aureus* strains produce PBPs for the synthesis of bacterial cell walls [68]. In resistant strains, this surrogate PBP2a replaces the function of normal PBPs, causing low binding affinity to most β-lactam antibiotics, thereby conferring resistance to MRSA against most members of this class of antibiotics [65,69]. Consequently, peptidoglycan biosynthesis, a main component of the bacterial cell wall, continues, and the bacterium evades cell death and lysis despite exposure to previously inhibitory concentrations of β-lactams [70]. The resistance exhibited by MRSA is achieved by acquiring a gene cassette containing *mec*A (the gene responsible for methicillin resistance) that encodes the altered transpeptidase PBP2a [71]. Therefore, PBP2a presents a target for designing novel antimicrobials to combat MRSA by inhibiting bacterial cell wall biosynthesis [65]. Although the number of studies on the role of commercially available pyrazole moieties and their derivatives (e.g., celecoxib) in the inhibition of mutant PBP2a is limited, it has been previously demonstrated that pyrazole-based amides displayed strong docking results against PBPs [72]. In addition, the inhibitory activity of pyrazole derivatives on bacterial cell wall biosynthesis has also been reported [73]. In contrast to oxacillin, which has a weak affinity for PBP2a [65], celecoxib may, therefore, inhibit the production of PBP2a and allow the antibiotic to bind to the normal PBPs, which results in the inhibition of cell wall formation and causes bacterial cell death. Moreover, celecoxib’s antibacterial mode of action against *S. aureus* ATCC 29213 has been investigated using a macromolecular synthesis assay. The results showed that the drug primarily exerted dose-dependent inhibition of RNA, DNA, and protein synthesis. Furthermore, disruption of lipid synthesis was evident at higher MIC concentrations, probably as a secondary effect of the RNA and protein synthesis inhibition [39]. In the present study, it appeared that celecoxib could, in some cases, resensitize MRSA strains to become susceptible to oxacillin. Therefore, we hypothesize that the inhibition of PBP2a by celecoxib may be suggested as a possible mechanism for its synergistic antibacterial action with oxacillin against *S. aureus*. Nonetheless, more mechanistic studies, including computational or bioinformatic methods [74], will be necessary to clarify the possible mechanism of synergistic antistaphylococcal action of the celecoxib and oxacillin combination at both cellular and molecular levels.

Although celecoxib produced the strongest in vitro antistaphylococcal activity compared with other anti-inflammatory drugs tested in this study, its MICs were higher than those of commonly used antibiotics. For example, vancomycin, primarily used as an antibiotic monotherapy for treating MRSA-associated infections, caused a decline in MRSA isolates at a MIC of 1.5 mg/L [75]. Nonetheless, at a vancomycin MIC of ≥2 mg/L, a higher mortality rate has been reported in adults with infections caused by MSSA [76]. On the other hand, the topical application of celecoxib significantly lowered the bacterial burden in a mouse model of MRSA skin infection, which suggests its greater in vivo efficacy [39]. Data from previous experiments, therefore, suggest celecoxib and its derivatives, which demonstrated a high antibacterial effect (MIC ≤ 2 mg/L) against *S. aureus* and *S. epidermidis*, as prospective candidates for developing agents with dual anti-inflammatory and antibacterial actions for the treatment of MI [38]. In the present study, celecoxib also produced a strong synergistic effect when combined with oxacillin, which proposes both agents for combined therapeutical use. Since the oral administration of celecoxib demonstrated an effect in minimizing post-operative inflammation and pain associated with arthroplasty [77], coadministration of celecoxib with antibiotics could increase the efficacy of preoperative antibiotic prophylaxis. Nonetheless, it is necessary to mention that a much higher concentration of celecoxib than those typically achieved in patients during the use of this agent as an anti-inflammatory drug would be required. For example, a previous clinical report showed that the peak plasma concentrations of celecoxib in humans were between 0.6 and 0.9 mg/L (600 and 900 ng/mL) following a single dose of 200 mg. Also, celecoxib is highly protein-bound, with about 97% of the drug being bound in vivo at clinical concentrations [78]. However, other drug delivery systems commonly used in medical practice may be considered [19,79]. Implants allow drugs to diffuse slowly in a more targeted approach, leading to controlled and sustained drug release from the implants to the site of infection in the body [19]. Since commercially available bone cement (e.g., PALACOS R + G, Heraeus Medical, Wehrheim, Germany) contains active ingredients (e.g., antibiotics) in percentage amounts, celecoxib can theoretically be incorporated into this therapeutical form in higher concentrations [80]. Therefore, implantable drug delivery systems may be an alternative to the coadministration of celecoxib and antibiotics. Due to the high concentration of celecoxib obtained in the present study, systemic administration is not considered to be the most appropriate pharmaceutical form for administering this drug combination. Instead, it can be proposed to incorporate celecoxib and oxacillin into a local delivery carrier (e.g., bone cement) in which the concentrations of antibiotics vary from 850 to 4000 mg/L [81,82,83]. This approach has the benefit of providing high local drug concentrations while minimizing systemic side effects by ensuring the local delivery of the agents in the infection sites.

With the aim of simulating the conditions of MI [84], the *S. aureus* strains were incubated at 37 °C for 24 h in the present study. This approach corresponds with other studies investigating the antimicrobial effect of celecoxib against *S. aureus* strains, including the clinical isolates resistant to methicillin and oxacillin [39]. In addition, previous studies show that most MRSA strains are becoming sensitive to temperatures higher than or equal to 40 °C [85,86]. Moreover, the final pH of the MHB was slightly increased to 7.6 to enhance the dissolution rate of celecoxib, as previous reports showed that the aqueous solubility of the drug increases at pHs ranging from 6.8 to 12, with a very high rate of solubility recorded at pH 12 [87,88]. Nevertheless, it is necessary to note that the modified experimental conditions (e.g., higher pH and temperature) may impact the results and data interpretation, especially related to MRSA strains. Also, the results of the present study must be confirmed in animal studies and clinical trials prior to the use of a combination of these drugs in clinical practice.

## 4. Materials and Methods

### 4.1. Chemicals

The anti-inflammatory drugs, namely aspirin, acemetacin, celecoxib, diclofenac sodium, diflunisal, diacerein, ibuprofen, nabumetone, and sulindac, which are used in treating patients with rheumatoid arthritis and osteoarthritis, were primarily selected for this study [55,89,90,91,92]. With the aim of comparing their effects with representatives of other classes of anti-inflammatory drugs, ampyrone, ethenzamide, felbinac, mefenamic acid, propyphenazone, sulindac sulfide, tolfenamic, and cortisone were included in the panel of tested agents. All anti-inflammatory drugs were obtained from Sigma-Aldrich (Prague, Czech Republic). The stock solutions of the anti-inflammatory agents were prepared using dimethyl sulfoxide (DMSO, Penta, Prague, Czech Republic) and deionized water for oxacillin. 

### 4.2. Staphylococcal Strains and Growth Media

In this study, ten *S. aureus* strains, including antibiotic-resistant and -susceptible forms, were tested. American Type Culture Collection (ATCC) standard strains 25923, 29213, 33591, 33592, 43300, and BAA 976 were purchased from Oxoid (Basingstoke, UK) on ready-to-use bacteriological Culti-Loops. Clinical isolates (MRSA1, MRSA2, MRSA3, and MRSA4) obtained from patients admitted to the intensive care units of the Motol University Hospital (Prague, Czech Republic) were also provided. Identification of the clinical isolates was performed by matrix-assisted laser desorption/ionization time-of-flight mass spectrometry, as described in previous work [93]. Cation-adjusted Mueller–Hinton broth (MHB, Oxoid, Basingstoke, UK) supplemented with 2% NaCl was used as the cultivation and assay medium. The pH of the broth was equilibrated to a final value of pH 7.6 using Trizma base (Sigma-Aldrich, Prague, Czech Republic) to enhance the dissolution rate of celecoxib [87,88].

### 4.3. Minimum Inhibitory Concentration (MIC) Determination 

The in vitro growth-inhibitory activities of the tested compounds against *S. aureus* strains were evaluated following the broth microdilution method using 96-well microtiter plates according to a slightly modified protocol of the Clinical and Laboratory Standards Institute (CLSI) [39,62,94]. Before testing, the bacteria strains were subcultured in MHB media at 37 °C for 24 h. The subcultured bacterial density of 1.5 × 10^8^ CFU mg/L was adjusted by 0.5 McFarland standard using a Densi-La-Meter II instrument (Lachema, Brno, CZ) to inoculate the 96-well plates (5 μg/well). Assay microplate preparation and serial dilution of the tested compounds were performed with an automated pipetting platform Freedom EVO 100 equipped with a four-channel liquid handling system (Tecan, Mannedorf, Switzerland). The initial concentration of oxacillin was optimized based on the susceptibility of the bacteria strains tested (4 and 512 mg/L for susceptible and resistant strains, respectively), whereas that of all other agents tested in this study was 512 mg/L. The microplates were then inoculated with bacterial cultures and incubated at 37 °C for 24 h. Since the mean temperature previously recorded in the knee of patients with suspected septic arthritis was 37.93 °C [84], *S. aureus* was incubated at 37 °C to simulate the conditions of MI. Subsequently, the bacterial growth was first observed by visual inspection of the microtiter plates and subsequently measured spectrophotometrically using a Multimode Reader Cytation 3 (BioTek Instruments, Winooski, VT, USA) at 405 nm [62,95]. Matching values from both visual and spectrophotometric evaluation were regarded as the final MIC. The MICs were expressed as the lowest concentration that inhibited bacteria growth by ≥80% compared to the agent-free growth control [96]. The assay was performed as three independent experiments, each carried out in triplicate. The mode and median were used for the final MIC value calculation when the triplicate endpoints for the particular staphylococcal strains were within the two- and three-dilution ranges of the drug tested, respectively [97]. The highest concentration of DMSO present in the microtiter plates (1%) did not inhibit the bacterial growth of any of the tested strains. At this concentration, DMSO has been reported to not produce antibacterial synergistic effects with antibiotics [98]. *S. aureus* ATCC 29213 was used as a control strain for antibiotic susceptibility testing. Oxacillin was used as a marker of methicillin resistance (MIC ≥ 4 mg/L) [62].

### 4.4. Evaluation of the Combined Antistaphylococcal Effect

The combined effect of oxacillin and celecoxib against *S. aureus* was evaluated using the checkerboard assay according to FICIs. Among the methods used to detect in vitro synergy between compounds, the checkerboard assay and time–kill curve are the most widely used. The checkerboard assay is a well-established tool used to determine the antimicrobial effects of two compounds in combination and encourages a conservative interpretation of results [99,100]. On the other hand, the technical parameters and interpretation of results are not standardized for the time–kill assay [101], which may lead to false positives. Thus, to strictly adhere to the criteria for synergy evaluation, the checkerboard assay was chosen to test the synergistic interactions between oxacillin and celecoxib. In combination, eight two-fold serial dilutions of the oxacillin prepared in horizontal rows of the microtiter plate were cross-diluted vertically by eight two-fold serial dilutions of celecoxib. Assay microplate preparation and serial dilution for the bacteria growth were performed as described in the MIC determination section above. The combined effects of oxacillin (A) and celecoxib (B) were determined based on the sum of the fractional inhibitory concentration index (∑FICI) values, which was calculated according to the following parameters: ∑FIC = FIC_A_ + FIC_B_, where FIC_A_ = MIC_A_ (in the presence of B)/MIC_A_ (alone), and FIC_B_ = MIC_B_ (in the presence of A)/MIC_B_ (alone). The results were interpreted as follows: a synergistic effect if ΣFICI ≤ 0.5, no interaction if ΣFICI > 0.5–4, and antagonistic if ΣFICI > 4 [100]. The MIC values for celecoxib and oxacillin used for the FICs calculations are the average of the MICs obtained from three independent experiments performed in triplicate.

## 5. Conclusions

In conclusion, among the 17 anti-inflammatory drugs tested in this study, 6 of them, namely celecoxib, diacerein, diflunisal, mefenamic acid, sulindac sulfide, and tolfenamic acid, produced a certain degree of in vitro growth-inhibitory effects against *S. aureus*. In comparison with other active drugs, celecoxib demonstrated the strongest antistaphylococcal activity, which suggests its potential dual anti-inflammatory and antibiotic action. Its antibacterial properties have been confirmed in a subsequent series of experiments showing its synergistic effect against both standard strains and clinical isolates of *S. aureus*, including MRSA, when combined with oxacillin. According to our best knowledge, this is the first report of the combined antistaphylococcal effect of celecoxib and oxacillin. These findings suggest celecoxib and its combination with oxacillin as perspective agents for research focused on the development of novel therapies (e.g., dual-action and combination drugs) for MI caused by *S. aureus*. Nevertheless, to comprehensively assess the antistaphylococcal efficacy of celecoxib and its combination with oxacillin, experiments focused on the use of primary cultures and cell line models relevant to MI should be performed in the future.

## Figures and Tables

**Table 1:** Susceptibility of *S. aureus* to anti-inflammatory agents in vitro.

Compound	Minimum Inhibitory Concentration in (mg/L) and (µM)									
	Standard ATCC ^a^ Strains						Clinical Isolates			
	25923	29213	33591 ^b^	33592 ^b^	43300 ^b^	BAA 976 ^b^	MRSA1 ^b^	MRSA2 ^b^	MRSA3 ^b^	MRSA4 ^b^
Acetylsalicylic acid	-	-	-	-	-	-	-	-	-	-
Acemetacin	-	-	-	-	-	-	-	-	-	-
Ampyrone	-	-	-	-	-	-	-	-	-	-
Celecoxib	64 (168)	64 (168)	64 (168)	64 (168)	64 (168)	64 (168)	32 (84)	64 (168)	64 (168)	64 (168)
Cortisone	-	-	-	-	-	-	-	-	-	-
Diacerein	128 (347)	128 (347)	128 (347)	64 (174)	128 (347)	128 (347)	128 (347)	128 (347)	64 (174)	128 (347)
Diclofenac sodium	-	-	-	-	-	-	-	-	-	-
Diflunisal	512 (2046)	512 (2046)	512 (2046)	512 (2046)	512 (2046)	-	512 (2046)	512 (2046)	-	-
Ethenzamide	-	-	-	-	-	-	-	-	-	-
Felbinac	-	-	-	-	-	-	-	-	-	-
Ibuprofen	-	-	-	-	-	-	-	-	-	-
Mefenamic acid	512 (2121)	512 (2121)	512 (2121)	512 (2121)	512 (2121)	-	-	512 (2121)	512 (2121)	-
Nabumetone	-	-	-	-	-	-	-	-	-	-
Propyphenazone	-	-	-	-	-	-	-	-	-	-
Sulindac sulfide	128 (376)	128 (376)	128 (376)	128 (376)	128 (376)	128 (376)	128 (376)	128 (376)	128 (376)	128 (376)
Sulindac	-	-	-	-	-	-	-	-	-	-
Tolfenamic acid	256 (978)	256 (978)	256 (978)	256 (978)	256 (978)	256 (978)	256 (978)	512 (1957)	256 (978)	128 (489)
Oxacillin ^c^	0.25 (0.623)	0.5 (1.247)	512 (1276)	512 (1276)	256 (637)	64 (159)	512 (1276)	256 (637)	256 (637)	256 (637)

The minimum inhibitory concentrations are expressed as mode and median values for triplicate endpoints within the two- and three-dilution range, respectively. ^a^ American Type Culture Collection. ^b^ Methicillin-resistant *S. aureus*. ^c^ Positive antibiotic control. - absence of antimicrobial activity.

**Table 2:** In vitro growth-inhibitory activity of oxacillin and celecoxib combinations against *S. aureus*.

*S. aureus*	MICs ^a^ Alone		MICs of CX and OX Combinations and Related FICI ^b^											
	OX ^c^	CX ^d^	CX MIC 16		CX MIC 8		CX MIC 4		CX MIC 2		CX MIC 1		CX MIC 0.5	
			OX MIC	FICI	OX MIC	FICI	OX MIC	FICI	OX MIC	FICI	OX MIC	FICI	OX MIC	FICI
ATCC ^e^ 29213	0.5	60.444	0.225	0.716	0.189	0.510	0.166	0.399	0.194	0.421	0.180	0.377	0.180	0.369
ATCC 25923	0.25	56.888	0.230	1.204	0.263	1.196	0.166	0.736	0.152	0.646	0.125	0.517	0.090	0.369
ATCC 33591	512	49.777	3.555	0.328	64.888	0.287	138.890	0.351	192.000	0.415	231.110	0.471	234.670	0.468
ATCC 33592	512	56.888	2.222	0.285	5.110	0.150	12.777	**0.095**	26.888	**0.087**	40.888	**0.097**	40.888	**0.088**
ATCC 43300	227.56	53.333	2.000	0.308	44.222	0.344	88.000	0.461	156.444	0.725	156.444	0.706	-	-
BAA 976	64	64	55.999	1.124	39.999	0.749	42.666	0.729	32.000	0.531	32.000	0.515	32.000	0.507
MRSA1	512	53.333	199.560	0.689	216.111	0.572	231.111	0.526	202.666	0.433	181.333	0.372	181.333	0.363
MRSA2	256	64	114.000	0.695	213.333	0.958	241.777	1.006	241.777	0.975	241.777	0.960	241.777	0.952
MRSA3	256	64	88.666	0.596	135.111	0.652	142.222	0.618	156.444	0.642	156.444	0.626	156.444	0.618
MRSA4	256	64	55.555	0.467	117.333	0.583	142.222	0.618	156.444	0.642	156.444	0.626	156.444	0.618

The values are expressed as an average of three independent experiments conducted in triplicate, with a synergistic effect if FICI ≤ 0.5, no interaction if FICI > 0.5–4, and antagonistic if FICI > 4. The bolded FICIs represent the most potent synergistic interactions. ^a^ Minimum inhibitory concentration. ^b^ Fractional inhibitory concentration index. ^c^ Oxacillin. ^d^ Celecoxib. ^e^ American Type Culture Collection. - = not evaluated. All MIC units are in mg/L.

## Data Availability

Data underlying this article are available in the article.

## References

[1] Sakr A., Bregeon F., Mege J.L., Rolain J.M., Blin O. (2018). *Staphylococcus aureus* nasal colonisation: An update on mechanisms, epidemiology, risk factors, and subsequent infections. Front. Microbiol..

[2] Brouillette E., Goetz C., Droppa-Almeida D., Chamberland S., Jacques M., Malouin F. (2022). Secondary *Staphylococcus aureus* intramammary colonisation is reduced by non-aureus staphylococci exoproducts. Microbes Infect..

[3] Kavanagh N., Ryan J.E., Widaa A., Sexton G., Fennell J., O’Rourke S., Cahill K.C., Kearney C.J., O’Brien F.J., Kerrigan S.W. (2018). Staphylococcal osteomyelitis: Disease progression, treatment challenges, and future directions. Clin. Microbiol. Rev..

[4] Kock R., Becker K., Cookson B., van Gemert-Pijnen J.E., Harbarth S., Kluytmans J., Mielke M., Peters G., Skov R.L., Struelens M.J. (2010). Methicillin-resistant *Staphylococcus aureus* (MRSA): Burden of disease and control challenges in Europe. Euro Surveill..

[5] Davis J.S. (2005). Management of bone and joint infections due to *Staphylococcus aureus*. Intern. Med. J..

[6] Masters E.A., Ricciardi B.F., Bentley K.L.D.M., Moriarty T.F., Schwarz E.M., Muthukrishnan G. (2022). Skeletal infections: Microbial pathogenesis, immunity, and clinical management. Nat. Rev. Microbial..

[7] Lang R., Minion J., Skinner S., Wong A. (2018). Disseminated *Exophiala dermatitidis* causing septic arthritis and osteomyelitis. BMC Infect. Dis..

[8] Sommer T., Karsy M., Driscoll M.J., Jensen R.L. (2018). Varicella-zoster virus infection and osteomyelitis of the skull. World Neurosurg..

[9] Tong S.Y.C., Davis J.S., Eichenberger E., Holland T.L., Fowler V.G. (2015). *Staphylococcus aureus* infections: Epidemiology, pathophysiology, clinical manifestations, and management. Clin. Microbial. Rev..

[10] Ferrand J., El Samad Y., Brunschweiler B., Grados F., Dehamchia-Rehailia N., Sejourne A., Schmit J.L., Gabrion A., Fardellone P., Paccou J. (2016). Morbimortality in adult patients with septic arthritis: A three-year hospital-based study. BMC. Infect. Dis..

[11] Huang J.F., Wu Q.N., Zheng X.Q., Sun X.L., Wu C.Y., Wang X.B., Wu C.W., Wang B., Wang X.Y., Bergman M. (2020). The characteristics and mortality of osteoporosis, osteomyelitis, or rheumatoid arthritis in the diabetes population: A retrospective study. Int. J. Endocrinol..

[12] Walter N., Baertl S., Alt V., Rupp M. (2021). What is the burden of osteomyelitis in Germany? An analysis of inpatient data from 2008 through 2018. BMC Infect. Dis..

[13] Minguez S., Molinos S., Mateo L., Gimenez M., Mateu L., Cabello J., Olive A. (2015). Septic arthritis due to methicillin-resistant *Staphylococcus aureus* in adults. Reumatol. Clin..

[14] Abram S.G.F., Alvand A., Judge A., Beard D.J., Price A.J. (2020). Mortality and adverse joint outcomes following septic arthritis of the native knee: A longitudinal cohort study of patients receiving arthroscopic washout. Lancet Infect. Dis..

[15] Lew D.P., Waldvogel F.A. (2004). Osteomyelitis. Lancet.

[16] Stake S., Scully R., Swenson S., Lee D., Lee R., Sparks A., Pandarinath R. (2020). Repeat irrigation and debridement for patients with acute septic knee arthritis: Incidence and risk factors. J. Clin. Orthop. Trauma.

[17] Vowden K.R., Vowden P. (1999). Wound debridement part 2: Sharp techniques. J. Wound Care..

[18] Urish K.L., Cassat J.E. (2020). *Staphylococcus aureus* osteomyelitis: Bone, bugs, and surgery. Infect. Immun..

[19] Smith M., Roberts M., Al-Kassas R. (2022). Implantable drug delivery systems for the treatment of osteomyelitis. Drug Dev. Ind. Pharm..

[20] Dombrowski J.C., Winston L.G. (2008). Clinical failures of appropriately treated methicillin-resistant *Staphylococcus aureus* infections. J. Infect..

[21] Wieland B.W., Marcantoni J.R., Bommarito K.M., Warren D.K., Marschall J. (2012). A retrospective comparison of ceftriaxone versus oxacillin for osteoarticular infections due to methicillin-susceptible *Staphylococcus aureus*. Clin. Infect. Dis..

[22] Woods C.R., Bradley J.S., Chatterjee A., Copley L.A., Robinson J., Kronman M.P., Arrieta A., Fowler S.L., Harrison C., Carrillo-Marquez M.A. (2021). Clinical practice guideline by the pediatric infectious diseases society and the infectious diseases society of America: 2021 guideline on diagnosis and management of acute hematogenous osteomyelitis in pediatrics. J. Pediatric. Infect. Dis. Soc..

[23] Stevens D.L. (2006). The role of vancomycin in the treatment paradigm. Clin. Infect. Dis..

[24] Liu C., Chambers H.F. (2003). *Staphylococcus aureus* with heterogeneous resistance to vancomycin: Epidemiology, clinical significance, and critical assessment of diagnostic methods. Antimicrob. Agents Chemother..

[25] Marinho D.S., Huff G., Ferreira B.L., Castro H., Rodrigues C.R., de Sousa V.P., Cabral L.M. (2011). The study of vancomycin use, and its adverse reactions associated to patients of a Brazilian university hospital. BMC Res. Notes.

[26] Thomas C., Stevenson M., Riley T.V. (2003). Antibiotics, and hospital-acquired *Clostridium difficile*-associated diarrhoea: A systematic review. J. Antimicrob. Chemother..

[27] Stevens D.L., Bisno A.L., Chambers H.F., Dellinger E.P., Goldstein E.J., Gorbach S.L., Hirschmann J.V., Kaplan S.L., Montoya J.G., Wade J.C. (2014). Clinical practice guidelines for the diagnosis and management of skin and soft tissue infections: 2014 Update by IDSA. Clin. Infect. Dis..

[28] Bell J.M., Turnidge J.D., Sentry A. (2002). High prevalence of oxacillin-resistant *Staphylococcus aureus* isolates from hospitalised patients in Asia-Pacific and South Africa: Results from sentry antimicrobial surveillance programme, 1998–1999. Antimicrob. Agents Chemother..

[29] Helito C.P., Zanon B.B., Miyahara H.D.E.S., Pecora J.R., Lima A.L., Oliveira P.R., Vicente J.R., Demange M.K., Camanho G.L. (2015). Clinical and epidemiological differences between septic arthritis of the knee and hip caused by oxacillin-sensitive and-resistant *Staphylococcus aureus*. Clinics.

[30] Sun W., Sanderson P.E., Zheng W. (2016). Drug combination therapy increases successful drug repositioning. Drug Discov. Today.

[31] Fischbach M.A. (2011). Combination therapies for combating antimicrobial resistance. Curr. Opin. Microbiol..

[32] Ruddaraju L.K., Pammi S.V.N., Guntuku G.S., Padavala V.S., Kolapalli V.R.M. (2020). A review on antibacterial to combat resistance: From the ancient era of plants and metals to present and future perspectives of green nanotechnological combinations. Asian J. Pharm. Sci..

[33] Toews M.L., Bylund D.B. (2005). Pharmacologic principles for combination therapy. Proc. Am. Thorac. Soc..

[34] Domingos O.D.S., Alcantara B.G.V., Santos M.F.C., Maiolini T.C.S., Dias D.F., Baldim J.L., Lago J.H.G., Soares M.G., Chagas-Paula D.A. (2019). Anti-inflammatory derivatives with dual mechanism of action from the metabolomic screening of *Poincianella pluviosa*. Molecules.

[35] Buchman A.L. (2001). Side effects of corticosteroid therapy. J. Clin. Gastroenterol..

[36] Williams D.M. (2018). Clinical pharmacology of corticosteroids. Respir. Care.

[37] Dogan A., Otlu S., Celebi O., Aksu P., Saglam A.G., Dogan A.N.C., Mutlu N. (2017). An investigation of antibacterial effects of steroids. Turkish J. Vet. Anim. Sci..

[38] Chiu H.C., Lee S.L., Kapuriya N., Wang D., Chen Y.R., Yu S.L., Kulp S.K., Teng L.J., Chen C.S. (2012). Development of novel antibacterial agents against methicillin-resistant *Staphylococcus aureus*. Bioorg. Med. Chem..

[39] Thangamani S., Younis W., Seleem M.N. (2015). Repurposing celecoxib as a topical antimicrobial agent. Front. Microbiol..

[40] Zhang S., Qu X., Tang H., Wang Y., Yang H., Yuan W., Yue B. (2021). Diclofenac resensitises methicillin-resistant *Staphylococcus aureus* to β-lactams and prevents implant infections. Adv. Sci..

[41] Kivitz A.J., Espinoza L.R., Sherrer Y.R., Liu-Dumaw M., West C.R.A. (2007). Comparison of the efficacy and safety of celecoxib 200 mg and celecoxib 400 mg once daily in treating the signs and symptoms of psoriatic arthritis. Semin. Arthritis Rheum..

[42] Tai F.W.D., McAlindon M.E. (2021). Non-steroidal anti-inflammatory drugs and the gastrointestinal tract. Clin. Med..

[43] Fitz Gerald G.A. (2003). COX-2 and beyond: Approaches to prostaglandin inhibition in human disease. Nat. Rev. Drug Discov..

[44] Howes L.G. (2007). Selective COX-2 inhibitors, NSAIDs, and cardiovascular events—Is celecoxib the safest choice?. Ther. Clin. Risk Manag..

[45] Silverstein F.E., Faich G., Goldstein J.L., Simon L.S., Pincus T., Whelton A., Makuch R., Eisen G., Agrawal N.M., Stenson W.F. (2000). Gastrointestinal toxicity with celecoxib vs nonsteroidal anti-inflammatory drugs for osteoarthritis and rheumatoid arthritis: The class study: A randomised controlled trial. Celecoxib long-term arthritis safety study. JAMA.

[46] Krasselt M., Baerwald C., Petros S., Seifert O. (2021). Mortality of sepsis in patients with rheumatoid arthritis: A single-center retrospective analysis and comparison with a control group. J. Intensive Care Med..

[47] Dinescu S.C., Barbulescu A.L., Firulescu S.C., Chisalau A.B., Parvanescu C.D., Ciurea P.L., Sandu R.E., Turcu-Stiolica A., Boldeanu M.V., Vintila E.M. (2021). *Staphylococcus aureus*-induced septic arthritis of the ankle related to malum perforans in a diabetes patient. Rom. J. Morphol. Embryol..

[48] Nugrahani I., Herawati D., Wibowo M.S. (2023). The benefits and challenges of antibiotics-non-steroidal anti-inflammatory drugs non-covalent reaction. Molecules.

[49] Kudva A., Kamath A.T., Dhara V., Ravindranath V. (2019). Chronic recurrent osteomyelitis: A surgeon’s enigma. J. Oral. Pathol. Med..

[50] Chan E.W.L., Yee Z.Y., Raja I., Yap J.K.Y. (2017). Synergistic effect of non-steroidal anti-inflammatory drugs (NSAIDs) on antibacterial activity of cefuroxime and chloramphenicol against methicillin-resistant *Staphylococcus aureus*. J. Glob. Antimicrob. Resist..

[51] Wald-Dickler N., Holtom P., Spellberg B. (2018). Busting the myth of “static vs cidal”: A systemic literature review. Clin. Infect. Dis..

[52] Pankey G.A., Sabath L.D. (2004). Clinical relevance of bacteriostatic versus bactericidal mechanisms of action in treating gram-positive bacterial infections. Clin. Infect. Dis..

[53] Bonnaire A., Vernet-Garnier V., Lebrun D., Bajolet O., Bonnet M., Hentzien M., Ohl X., Diallo S., Bani-Sadr F. (2021). Clindamycin combination treatment for the treatment of bone and joint infections caused by clindamycin-susceptible, erythromycin-resistant *Staphylococcus* spp.. Diagn. Microbiol. Infect. Dis..

[54] Fidelix T.S., Macedo C.R., Maxwell L.J., Fernandes Moca Trevisani V. (2014). Diacerein for osteoarthritis. Cochrane Database Syst. Rev..

[55] Pavelka K., Bruyere O., Cooper C. (2016). Diacerein: Benefits, risks, and place in the management of osteoarthritis. An opinion-based report from the ESCEO. Drugs Aging.

[56] Nguon S., Novy P., Kokoska L. (2013). Potentiation of the in vitro antistaphylococcal effect of oxacillin and tetracycline by the anti-inflammatory drug diacetyl rhein. Chemotherapy.

[57] Zhang H., Liu S., Yue J., Sun S., Lv Q., Jian S., Xie Y., Han L., Zhang F., Dai Y. (2019). In vitro antimicrobial activity of diacerein on 76 isolates of gram-positive cocci from bacterial keratitis patients and in vivo study of diacerein eye drops on *Staphylococcus aureus* keratitis in mice. Antimicrob. Agents Chemother..

[58] Seong Y.J., Alhashimi M., Mayhoub A., Mohammad H., Seleem M.N. (2020). Repurposing fenamic acid drugs to combat multidrug resistant *Neisseria gonorrhoeae*. Antimicrob. Agents Chemother..

[59] Yin Z., Wang Y., Whittell L.R., Jergic S., Liu M., Harry E., Dixon N.E., Kelso M.J., Beck J.L., Oakley A.J. (2014). DNA replication is the target for the antibacterial effects of nonsteroidal anti-inflammatory drugs. Chem. Biol..

[60] Etienne F., Resnick L., Sagher D., Brot N., Weissbach H. (2003). Reduction of sulindac to its active metabolite, sulindac sulfide: Assay and role of the methionine sulfoxide reductase system. Biochem. Biophys. Res. Commun..

[61] Shirin H., Moss S.F., Kancherla S., Kancherla K., Holt P.R., Weinstein I.B., Sordillo E.M. (2006). Nonsteroidal anti-inflammatory drugs have bacteriostatic and bactericidal activity against *Helicobacter pylori*. J. Gastroenterol. Hepatol..

[62] Clinical and Laboratory Standards Institute (2015). Methods for Dilution Antimicrobial Susceptibility Tests for Bacteria That Grow Aerobically Approved Standard.

[63] Annamanedi M., Kalle A.M. (2014). Celecoxib sensitises *Staphylococcus aureus* to antibiotics in macrophages by modulating SIRT1. PLoS ONE.

[64] Annamanedi M., Varma G.Y.N., Anuradha K., Kalle A.M. (2017). Celecoxib enhances the efficacy of low-dose antibiotic treatment against polymicrobial sepsis in mice and clinical Isolates of ESKAPE pathogens. Front. Microbiol..

[65] Shalaby M.W., Dokla E.M.E., Serya R.A.T., Abouzid K.A.M. (2020). Penicillin binding protein 2a: An overview and a medicinal chemistry perspective. Eur. J. Med. Chem..

[66] Santiago C., Pang E.L., Lim K.H., Loh H.S., Ting K.N. (2015). Inhibition of penicillin-binding protein 2a (PBP2a) in methicillin-resistant *Staphylococcus aureus* (MRSA) by combination of ampicillin and a bioactive fraction from *Duabanga grandiflora*. MC Complement. Altern. Med..

[67] Peacock S.J., Paterson G.K. (2015). Mechanisms of methicillin resistance in *Staphylococcus aureus*. Annu. Rev. Biochem..

[68] Santiago C., Pang E.L., Lim K.H., Loh H.S., Ting K.N. (2014). Reversal of ampicillin resistance in MRSA via inhibition of penicillin-binding protein 2a by *Acalypha wilkesiana*. BioMed. Res. Int..

[69] Zhou T., Li Z., Kang O.H., Mun S.H., Seo Y.S., Kong R., Shin D.W., Liu X.Q., Kwon D.Y. (2017). Antimicrobial activity, and synergism of ursolic acid 3-O-α-L-Arabinopyranoside with oxacillin against methicillin-resistant *Staphylococcus aureus*. Int. J. Mol. Med..

[70] Pinho M.G., Filipe S.R., de Lencastre H., Tomasz A. (2001). Complementation of the essential peptidoglycan transpeptidase function of penicillin-binding protein 2 (PBP2) by the drug resistance protein PBP2A in *Staphylococcus aureus*. J. Bacteriol..

[71] Fuda C., Suvorov M., Vakulenko S.B., Mobashery S. (2004). The basis for resistance to beta-lactam antibiotics by penicillin-binding protein 2a of methicillin-resistant *Staphylococcus aureus*. J. Biol. Chem..

[72] Sadeghian H., Sadeghian A., Pordel M., Rahimizadeh M., Jahandari P., Orafaie A., Bakavoli M. (2010). Design, synthesis, and structure–activity relationship study of 5-amido-1-(2,4-dinitrophenyl)-1*H*-4-pyrazolecarbonitrils as DD-carboxypeptidase/penicillin-binding protein inhibitors with Gram-positive antibacterial activity. Med. Chem. Res..

[73] Li Z., Francisco G.D., Hu W., Labthavikul P., Petersen P.J., Severin A., Singh G., Yang Y., Rasmussen B.A., Lin Y. (2003). 2-Phenyl-5,6-dihydro-2H-thieno[3,2-c]pyrazol-3-ol derivatives as new inhibitors of bacterial cell wall biosynthesis. Bioorg. Med. Chem. Lett..

[74] Preuer K., Lewis R.P.I., Hochreiter S., Bender A., Bulusu K.C., Klambauer G. (2018). DeepSynergy: Predicting anti-cancer drug synergy with deep Learning. Bioinformatics.

[75] Kok E.Y., Vallejo J.G., Sommer L.M., Rosas L., Kaplan S.L., Hulten K.G., McNeil J.C. (2018). Association of vancomycin MIC and molecular characteristics with clinical outcomes in methicillin-susceptible *Staphylococcus aureus* acute hematogenous osteoarticular infections in children. Antimicrob. Agents Chemother..

[76] Holmes N.E., Turnidge J.D., Munckhof W.J., Robinson J.O., Korman T.M., O’Sullivan M.V., Anderson T.L., Roberts S.A., Gao W., Christiansen K.J. (2011). Antibiotic choice may not explain poorer outcomes in patients with *Staphylococcus aureus* bacteremia and high vancomycin minimum inhibitory concentrations. J. Infect. Dis..

[77] Xu J., Li H., Zheng C., Zheng C., Wang B., Shen P., Xie Z., Qu Y. (2019). Efficacy of pre-emptive use of cyclooxygenase-2 inhibitors for total knee arthroplasty: A mini-review. Arthroplasty.

[78] FDA (1998). Centre for Drug Evaluation and Research: Application Number NDA 20-998.

[79] Sidney L.E., Heathman T.R., Britchford E.R., Abed A., Rahman C.V., Buttery L.D. (2015). Investigation of localized delivery of diclofenac sodium from poly (D, L-lactic acid-co-glycolic acid)/poly (ethylene glycol) scaffolds using an in vitro osteoblast inflammation model. Tissue Eng. Part A.

[80] Heraeus Medical Palacos R+G: High-Viscosity, Bone Cement With Gentamicin; Heraeus Medical GmbH, Germany. https://www.heraeus-medical.com/en/healthcare-professionals/products/palacos-rg/.

[81] Humez M., Domann E., Thormann K.M., Folsch C., Strathausen R., Vogt S., Alt V., Kuhn K.D. (2023). Daptomycin-impregnated PMMA cement against vancomycin-resistant germs: Dosage, handling, elution, mechanical stability, and effectiveness. Antibiotics.

[82] PRO-IMPLANT Foundation (2018). Pocket Guide to Diagnosing and Treating the Periprosthetic Joint Infection (PJI).

[83] Gogia J.S., Meehan J.P., Di Cesare P.E., Jamali A.A. (2009). Local antibiotic therapy in osteomyelitis. Semin. Plast. Surg..

[84] Gunay H., Bakan O.M., Mirzazade J., Sozbilen M.C. (2023). A new perspective on the diagnosis of septic arthritis: High-resolution thermal imaging. J. Clin. Med..

[85] Cunha B.A. (2005). Methicillin-resistant *Staphylococcus aureus*: Clinical manifestations and antimicrobial therapy. Microbiol. Infect..

[86] Missiakas D.M., Schneewind O. (2013). Growth and laboratory maintenance of *Staphylococcus aureus*. Curr. Protoc. Microbiol..

[87] Dolenc A., Kristl J., Baumgartner S., Planinsek O. (2009). Advantages of celecoxib nanosuspension formulation and transformation into tablets. Int. J. Pharm..

[88] Arslan A., Yet B., Nemutlu E., Akdag Y.C., Eroglu H., Oner L. (2023). Celecoxib Nanoformulations with Enhanced solubility, dissolution rate, and oral bioavailability: Experimental approaches over in vitro/in vivo evaluation. Pharmaceutics.

[89] John Hopkins Arthritis Centre (2024). Rheumatoid Arthritis Treatment.

[90] Crofford L.J. (2013). Use of NSAIDs in treating patients with arthritis. Arthritis Res. Ther..

[91] Moore R.A., Derry S., McQuay H.J. (2009). Single dose oral acemetacin for acute postoperative pain in adults. Cochrane Database Syst. Rev..

[92] Ahmed S., Sheraz M.A., Ahmad I. (2018). Tolfenamic Acid. Profiles Drug Subst. Excip. Relat. Methodol..

[93] Rondevaldova J., Hummelova J., Tauchen J., Kokoska L. (2018). In vitro antistaphylococcal synergistic effect of isoflavone metabolite demethyltexasin with amoxicillin and oxacillin. Microb. Drug Resist..

[94] Mohamed M.F., Hamed M.I., Panitch A., Seleem M.N. (2014). Targeting methicillin-resistant *Staphylococcus aureus* with short salt-resistant synthetic peptides. Antimicrob. Agents Chemother..

[95] Cos P., Vlietinck A.J., Berghe D.V., Maes L. (2006). Anti-infective potential of natural products: How to develop a stronger in vitro proof-of-concept. J. Ethnopharmacol..

[96] Jorgensen J.H., Turnidge J.D., Washington J.A. (1999). Antibacterial susceptibility tests: Dilution and disk diffusion methods. Manual of Clinical Microbiology, 7th ed Murray, P.R., Baron, E.J., Pfaller, M.A., Tenover, F.C., Yolken, R.H., Eds..

[97] Frankova A., Vistejnova L., Merinas-Amo T., Leheckova Z., Doskocil I., Wong Soon J., Kudera T., Laupua F., Alonso-Moraga A., Kokoska L. (2021). In vitro antibacterial activity of extracts from Samoan medicinal plants and their effect on proliferation and migration of human fibroblasts. J. Ethnopharmacol..

[98] Summer K., Browne J., Hollanders M., Benkendorff K. (2022). Out of control: The need for standardised solvent approaches and data reporting in antibiofilm assays incorporating dimethyl-sulfoxide (DMSO). Biofilm.

[99] White R.L., Burgess D.S., Manduru M., Bosso J. (1996). Comparison of three different in vitro methods of detecting synergy: Time-kill, checkerboard, and E-test. Antimicrob. Agents Chemother..

[100] Odds F.C. (2003). Synergy, antagonism, and what the chequerboard puts between them. J. Antimicrob. Chemother..

[101] Bidaud A.L., Schwarz P., Herbreteau G., Dannaoui E. (2022). Techniques for the assessment of in vitro and in vivo antifungal combinations. J. Fungi.

